# Integrating blood biochemistry and plasma metabolomics to define age-related metabolic biomarkers in Alope village chickens

**DOI:** 10.14202/vetworld.2026.619-630

**Published:** 2026-02-17

**Authors:** Andi Mujnisa, Athhar Manabi Diansyah, Muhammad Ridwan Bahar, Andi Muhammad Fuad Al Kautsar Walinono, Aji Praba Baskara, Aeni Nurlatifah, Herdis Herdis, Fitra Aji Pamungkas, Syahruddin Said

**Affiliations:** 1 Department of Animal Nutrition, Faculty of Animal Science, Hasanuddin University, Jl. Perintis Kemerdekaan Km. 10 Tamalanrea Makassar, South Sulawesi, Indonesia.; 2 Department of Animal Production, Faculty of Animal Science, Hasanuddin University, Jl. Perintis Kemerdekaan Km. 10 Tamalanrea Makassar, South Sulawesi, Indonesia.; 3 Faculty of Vocational Study, Jl. Perintis Kemerdekaan Km. 10 Tamalanrea Makassar, South Sulawesi, Indonesia.; 4 Department of Nutrition and Feed Technology, Faculty of Animal Science, Gadjah Mada University, Depok, Sleman Regency, Special Region of Yogyakarta, Indonesia.; 5 Research Center for Animal Husbandry, National Research and Innovation Agency, Raya Jakarta, Bogor, West Java 16915, Indonesia; 6 Research Center for Applied Zoology, National Research and Innovation Agency, Cibinong Science Center, Jl. Raya Jakarta, Bogor, West Java 16915, Indonesia

**Keywords:** age-related biomarkers, Alope village chicken, blood biochemistry, *Gallus gallus domesticus*, GC–MS metabolomics, growth physiology, plasma metabolomics, precision nutrition

## Abstract

**Background and Ai:**

The Alope village chicken is an indigenous Indonesian line developed to combine local adaptability with improved growth potential. However, age-specific physiological and metabolic reference data remain limited, constraining evidence-based feeding and health management. This study aimed to integrate blood biochemical profiling and plasma metabolomics to characterize age-related metabolic changes, establish baseline reference values, and identify putative age-discriminant biomarkers in Alope village chickens.

**Materials and Methods:**

A cross-sectional design was applied using 30 clinically healthy female Alope village chickens (*Gallus gallus domesticus*), stratified into three age groups: G1 (8 weeks), G2 (12 weeks), and G3 (16 weeks) (n = 10 per group). Blood samples were collected for biochemical analysis of glucose, total protein, urea, and triglycerides using enzymatic colorimetric assays. Plasma metabolomic profiling was conducted using untargeted gas chromatography–mass spectrometry. Multivariate analyses, including principal component analysis and partial least squares–discriminant analysis, were used to explore age-associated metabolic patterns. Discriminant metabolites were identified based on variable importance in projection values and descriptive receiver operating characteristic analysis.

**Result:**

All biochemical parameters increased significantly with age (p < 0.05), remaining within physiological reference ranges, indicating normal metabolic development. Multivariate metabolomic analysis revealed clear age-dependent separation, with distinct metabolic signatures characterizing each growth stage. Exploratory pathway mapping indicated age-associated involvement of amino acid metabolism (glycine, serine, and threonine), lipid metabolism (glycerolipid and glycerophospholipid turnover), purine metabolism, the citrate cycle, and nodes linked to the pentose phosphate pathway. Seven metabolites demonstrated strong discriminatory performance (variable importance in projection ≈ 1.17–1.35; area under the curve ≥ 0.985) and were identified as putative age-related biomarkers, reflecting coordinated shifts in one-carbon/redox balance, membrane remodeling, and nucleotide and energy metabolism.

**Conclusio:**

Age was the primary determinant of metabolic organization in Alope village chickens from 8 to 16 weeks. The integration of blood biochemistry and plasma metabolomics revealed coordinated physiological transitions from early growth toward enhanced energy handling and metabolic stabilization. The identified candidate metabolites provide an exploratory framework for defining physiological age and support the potential application of age-informed metabolic indicators for precision nutrition and health monitoring in indigenous village chicken production systems.

## INTRODUCTION

The Alope variety of village chickens was developed to integrate the environmental resilience typical of Indonesian backyard poultry with improved growth performance and economic value under smallholder production systems [1]. Despite its increasing adoption, age-resolved physiological baselines specific to this variety remain limited, thereby constraining evidence-based ration formulation, health monitoring, and breeding decisions [2, 3]. In village chickens, growth from the early grower to pre-adult stages is characterized by coordinated changes in tissue accretion, organ functional maturation, and metabolic efficiency, which progressively modify nutrient requirements [4]. In practical production systems, the ages of 8, 12, and 16 weeks represent biologically meaningful decision points, corresponding to early growth (rapid muscle accretion and high protein demand), mid-growth transition (shifts in energy partitioning and metabolic flexibility), and late grower stages approaching physiological maturity, during which lipid deposition and metabolic homeostasis become increasingly prominent [4].

Blood biochemistry provides a concise overview of these ontogenetic changes. Circulating glucose reflects the balance between energy provision and utilization through glycolytic and gluconeogenic pathways; total protein serves as an index of nutritional adequacy and hepatic synthetic capacity; urea, although present at low concentrations in birds, remains a useful indicator of overall nitrogen turnover and amino acid catabolism; and triglycerides reflect lipid mobilization and hepatic lipoprotein packaging [5, 6]. Collectively, these markers are expected to change systematically with age, mirroring a shift in substrate allocation from anabolic tissue accretion toward enhanced management of energy reserves and more stable metabolic homeostasis at later growth stages [7].

Metabolomics complements these composite indices by resolving pathway-level mechanisms underlying metabolic adaptation. Mapping the plasma small-molecule spectrum to curated databases such as Human Metabolome Database (HMDB) and Kyoto Encyclopedia of Genes and Genomes (KEGG) enables the identification of coordinated metabolic modules, commonly involving amino acid metabolism (including glycine, serine, and threonine, as well as branched-chain amino acids), carbohydrate–lipid interfaces such as glycerolipid and glycerophospholipid metabolism, purine turnover associated with nucleotide economy and redox buffering, and contributions from the pentose phosphate pathway that supply nicotinamide adenine dinucleotide phosphate (NADPH) for biosynthesis and oxidative defense [8, 9]. Importantly, outputs from pathway enrichment analyses should be interpreted as topological representations of shared metabolic nodes rather than evidence of direct pathway activation and therefore require careful contextualization within avian physiology [10].

Unlike previous poultry metabolomics studies that have largely focused on commercial broilers, breeder stocks, or experimental lines, the present study establishes physiological age stratification in an indigenous village chicken using an integrated clinical biochemistry–metabolomics approach. To date, comparable integrative metabolomic investigations in village or indigenous chicken populations remain scarce, highlighting a clear knowledge gap in poultry systems relevant to smallholder and One Health contexts [2, 11]. Accordingly, this study was designed as a hypothesis-driven, cross-sectional investigation, based on the premise that both classical blood biochemistry and pathway-level plasma metabolomic profiles would consistently reflect age-associated metabolic reprogramming in *Gallus gallus domesticus*. By addressing this gap, the study aims to generate age-specific biochemical and metabolomic reference baselines and to nominate candidate biomarkers that may support precision nutrition, health surveillance, and sustainable improvement strategies for smallholder production systems of Alope chickens (*G. g. domesticus*) [12].

Although blood biochemical profiling and metabolomics have been increasingly applied to poultry research, most existing studies have focused on commercial broilers, breeder stocks, or experimentally selected lines, with limited relevance to indigenous or village chicken populations. For Alope chickens, age-specific physiological reference values remain poorly defined, particularly those integrating classical blood biochemistry with pathway-level plasma metabolomic information. Available reports generally provide isolated biochemical ranges or omics-based snapshots without explicitly linking metabolic organization to biologically meaningful growth stages. Moreover, integrative metabolomic investigations in village chickens remain scarce, creating a critical gap in understanding how metabolic reprogramming progresses with age in *G. g. domesticus* under smallholder-relevant conditions. This lack of age-resolved metabolic baselines constrains the development of precision feeding strategies, limits early physiological monitoring, and hampers the identification of robust metabolic indicators applicable to sustainable village chicken production and One Health–oriented systems.

Accordingly, the present study aimed to characterize age-associated metabolic reorganization in Alope chickens by integrating blood biochemical parameters with untargeted plasma metabolomic profiling across key growth stages. Specifically, this study sought to (i) establish age-specific reference patterns for glucose, total protein, urea, and triglycerides, (ii) describe age-dependent shifts in plasma metabolomic signatures and associated metabolic pathways, and (iii) nominate putative age-discriminant metabolites as candidate biomarkers reflecting physiological maturation in *G. g. domesticus*. By generating integrated biochemical and metabolomic baselines, this work aims to support age-informed precision nutrition, physiological monitoring, and sustainable improvement strategies for Alope chickens under smallholder production systems.

## MATERIALS AND METHODS

### Ethical approval

The experimental protocol, including handling and blood sampling, was approved by the Ethics Committee of the Faculty of Animal Science, Hasanuddin University (Approval No. 016/UN4.12/EC/VI/2025). Blood collection was performed by trained personnel using standard restraint to minimize stress, and all birds were monitored for adverse effects throughout the procedure, in compliance with applicable institutional and national animal welfare guidelines.

### Study period and location

The study was conducted from June to October 2025. Clinical chemistry analyses were performed at the Faculty of Animal Science, Universitas Hasanuddin (Makassar, Indonesia), and metabolomic profiling was conducted at the National Research and Innovation Agency (BRIN), Cibinong, West Java, Indonesia.

### Study design and experimental overview

A cross-sectional experimental design was employed to characterize age-related differences in blood biochemical parameters and plasma metabolomic profiles of Alope village chickens (G. g. domesticus). Thereafter, the species is referred to as *G. g. domesticus*. A total of 30 clinically healthy female birds were included and stratified into three biologically relevant age groups: G1 (8 weeks), G2 (12 weeks), and G3 (16 weeks), with n = 10 birds per group. Birds were reared under a semi-intensive floor housing system with litter, provided ad libitum access to feed and drinking water, and maintained under typical tropical environmental conditions. The primary objective was to conduct an age-wise comparison of classical biochemical indices and untargeted plasma metabolomic signatures to describe metabolic maturation during growth. Blood samples were collected in the morning before feeding, without prolonged fasting, to minimize postprandial variation while avoiding fasting-induced metabolic stress.

The analytical workflow integrated clinical chemistry assays, untargeted gas chromatography–mass spectrometry (GC–MS)–based plasma metabolomics, and univariate and multivariate statistical analyses to identify age-associated metabolic patterns and putative biomarker candidates.

### Housing, diet composition, and feeding management

Birds were fed a commercially formulated grower diet commonly used for village and slow-growing chickens under semi-intensive systems. Feed and clean drinking water were provided ad libitum throughout the experimental period. The same diet was offered to all age groups to ensure that observed metabolic differences primarily reflected age-related physiological variation rather than dietary effects. The diet contained approximately 18%–19% crude protein and 2,800–2,900 kcal/kg metabolizable energy on a dry matter basis, consistent with recommended nutrient specifications for growing chickens between 8 and 16 weeks of age. Feed was offered twice daily, and minimal refusals were observed, indicating consistent intake across groups.

### Sample collection

Blood was collected from the brachial (wing) vein of each bird in the morning before feeding. Two tubes were obtained per bird: a lithium-heparin tube for plasma intended for biochemical analysis and a dipotassium ethylenediaminetetraacetic acid (K_2_-EDTA) tube for plasma intended for metabolomic analysis. Tubes were gently inverted 8–10 times, kept on wet ice, and processed within 30 min. Plasma was separated by centrifugation at 1,500–2,000 × g for 10–15 min at 4°C, aliquoted into pre-chilled microtubes, immediately frozen, and stored at −80°C with a single freeze–thaw cycle maximum. Heparinized plasma was used for biochemical analyses, whereas EDTA plasma was used for metabolomic analyses [13].

### Biochemical analysis

Glucose, total protein, urea, and triglycerides were quantified in lithium-heparin plasma by enzymatic colorimetry using an automated clinical analyzer (cobas c 311, Roche Diagnostics, Mannheim, Germany) with manufacturer-supplied reagents and calibrators. When automation was unavailable, identical chemistries were performed in 96-well format using a microplate spectrophotometer (Multiskan SkyHigh, Thermo Fisher Scientific, Waltham, MA, USA). Assay principles included the hexokinase/glucose-6-phosphate dehydrogenase (G6PD) method for glucose (340 nm), the biuret method for total protein (540–560 nm), the urease–glutamate dehydrogenase ultraviolet method for urea (decrease of nicotinamide adenine dinucleotide, reduced form [NADH], at 340 nm), and the glycerol-3-phosphate oxidase–peroxidase (GPO–PAP) method for triglycerides (~500–550 nm, with glycerol blank where applicable).

Although uric acid (UA) is the primary nitrogenous waste product in birds, urea was included as a supportive indicator of general nitrogen turnover and amino acid catabolism, as commonly applied in avian biochemical profiling and comparative physiological studies. Each analytical batch included multilevel calibration and two-level internal quality controls (Liquichek, Bio-Rad Laboratories, Irvine, CA, USA). Samples were analyzed in duplicate, with intra-assay coefficients of variation <5% for glucose and total protein and <7% for urea and triglycerides. Samples exceeding predefined acceptance criteria were reanalyzed. Plasma was separated within 30 min of venipuncture and subjected to a single freeze–thaw cycle before analysis [14].

### Metabolomic analysis

#### Metabolite extraction and derivatization

An untargeted plasma metabolomics approach was employed. A 50 µL aliquot of plasma was transferred into a 1.5 mL microtube, followed by the addition of 150 µL methoxyamine hydrochloride in methanol (1 mg/mL) and 350 µL extraction solvent (ultrapure water:methanol, 1:4). Samples were vortexed for 1 min and centrifuged at 14,171 × *g* for 20 min at 4°C. The supernatant was filtered through a 25-mm syringe filter and transferred to a new microtube. Filtrates were evaporated at 36°C using a vacuum evaporator for approximately 2 h until complete dryness and stored at 20°C until derivatization [15].

For derivatization, 100 µL N-trimethylsilyl-N-methyl trifluoroacetamide with 1% trimethylchlorosilane (MSTFA + 1% TMCS) was added, followed by incubation at 70°C for 1 h. Samples were centrifuged again at 14,171 × *g* for 10 min at 4°C, and supernatants were transferred into amber glass GC vials. Two pooled quality control (QC) samples were prepared from equal volumes of all samples. Features with coefficients of variation >30% in QC samples were excluded, and QC-based signal correction and normalization were applied prior to multivariate analyses.

#### GC–MS analysis

Metabolomic profiling was performed using a GC–MS system (GCMS-QP2010, Shimadzu Co., Japan) equipped with an SH-RI-5MS capillary column (30 m × 0.25 mm × 0.25 µm). A 1 µL aliquot of each derivatized sample was injected. Injector, interface, and ion source temperatures were set at 270°C, 260°C, and 200°C, respectively. Helium (99.9%) was used as the carrier gas at a flow rate of 3 mL/min. The oven temperature program was set to hold at 80°C for 2 min, increase to 325°C at 10°C/min, and hold for 6 min. The solvent cut-off time was 3 min. Electron ionization was performed at 70 eV, and mass spectra were acquired over a range of 30–600 m/z.

Metabolite identification was performed by matching mass spectra against the National Institute of Standards and Technology (NIST) 20 mass spectral library and confirmed using retention index (RI) comparison derived from alkane standards. Identifications were assigned according to the Metabolomics Standards Initiative, with metabolites matched by both mass spectra and RI classified as Level 2 (putatively annotated compounds). Results are reported as relative abundances [16].

### Statistical analysis

Blood biochemical data were tested for normality using the Shapiro–Wilk test and analyzed using one-way analysis of variance (ANOVA), followed by Tukey’s honest significant difference post hoc test where appropriate. Results are presented as mean ± standard deviation, and statistical significance was set at p < 0.05. Raw GC–MS data were processed using MetaboAnalyst 6.0 for peak detection, deconvolution, and retention time alignment. Features with coefficients of variation >30% in QC samples or present in <50% of samples in at least one group were excluded. Data were normalized, log-transformed, and Pareto-scaled prior to analysis. Principal component analysis was used for exploratory visualization, and age-discriminant metabolites were identified using partial least squares–discriminant analysis. Model robustness was evaluated using 7-fold cross-validation and 200 permutation tests. Univariate metabolite differences were assessed using ANOVA with Benjamini–Hochberg false discovery rate correction. Metabolites with variable importance in projection >1.0 and adjusted p < 0.05 were considered discriminant, and receiver operating characteristic curve analysis was used to assess classification performance by area under the curve (AUC). Statistical analyses were conducted using SPSS version 26.0 (IBM Corp., Armonk, NY, USA), MetaboAnalyst 6.0, and GraphPad Prism 9.0 (GraphPad Software Inc., San Diego, CA, USA).

## RESULTS

### Blood biochemical parameters

**Figure 1:**
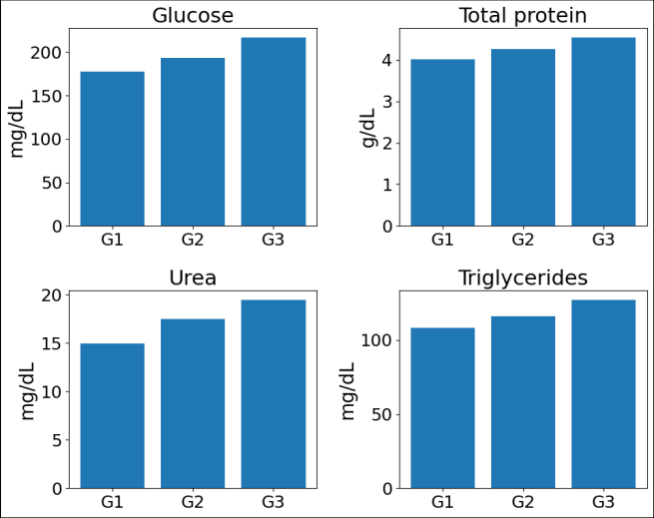
Age-related changes in blood biochemical parameters of Alope chickens across the 8–16-week growth period. Mean ± standard deviation values of glucose, total protein, urea, and triglycerides are shown for G1 (8 weeks), G2 (12 weeks), and G3 (16 weeks). Different superscript letters above bars within each panel indicate statistically significant differences among age groups (p < 0.05). The progressive increase in all parameters reflects coordinated metabolic maturation with age.

Four plasma biochemical parameters, namely glucose, total protein, urea, and triglycerides, were measured in Alope chickens across increasing ages (Figure 1). Glucose increased from 177.80 ± 10.32 mg/dL at 8 weeks to 193.30 ± 10.13 mg/dL at 12 weeks and 216.60 ± 10.78 mg/dL at 16 weeks, corresponding to relative gains of 8.72% (8→12 weeks) and 12.05% (12→16 weeks). Total protein increased from 4.01 ± 0.14 g/dL to 4.26 ± 0.14 g/dL and 4.54 ± 0.15 g/dL, representing increases of 6.23% and 6.57%, respectively. Urea concentrations increased from 14.94 ± 0.65 mg/dL to 17.50 ± 0.63 mg/dL and 19.43 ± 1.12 mg/dL (17.14% and 11.03%, respectively). Triglyceride levels increased from 108.10 ± 4.56 mg/dL to 115.80 ± 5.41 mg/dL and 127.00 ± 6.18 mg/dL, corresponding to relative increases of 7.12% and 9.67%. All biochemical parameters differed significantly among age groups (*p* < 0.05) and remained within published physiological reference ranges for growing chickens, indicating normal metabolic status throughout the study period. Effect size estimates (η²) indicated moderate to large age effects, particularly for glucose and triglycerides, underscoring the biological relevance of the observed age-associated changes.

### Differential metabolite profiles

**Figure 2:**
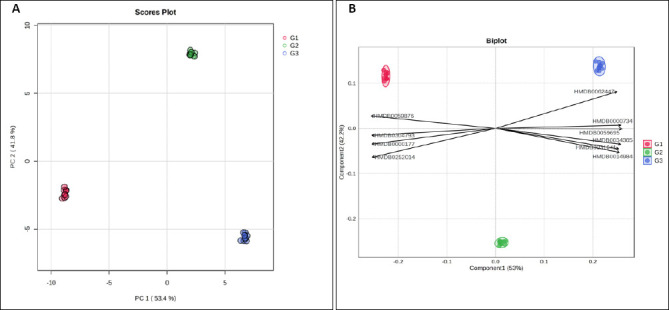
Multivariate analysis of plasma metabolites in Alope village chickens across age groups. (A) Principal component analysis score plot showing age-dependent clustering of samples. (B) Partial least squares–discriminant analysis biplot highlighting metabolites contributing to group separation.

Multivariate projection of the plasma metabolome revealed a clear age-dependent structure. The three age groups formed tight, non-overlapping clusters in the principal component analysis (PCA) score plot (Figure 2A), with G1 loading predominantly on negative PC1, G2 on positive PC2, and G3 on positive PC1/negative PC2, indicating marked between-group separation and limited within-group dispersion. Partial least squares–discriminant analysis (PLS-DA) analysis (Figure 2B) identified metabolites driving this separation, with several HMDB-annotated features aligning toward the G3 centroid, whereas others were associated with G1 or G2, consistent with progressive metabolic changes across growth stages.

**Figure 3:**
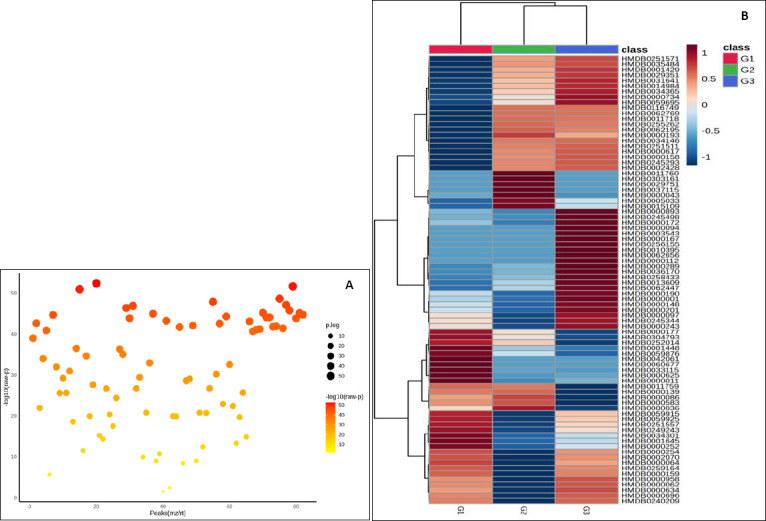
Differential metabolites in Alope village chickens across age groups. (A) Analysis of variance bubble plot showing metabolites with significant age-associated differences. (B) Hierarchical clustering heatmap illustrating age-dependent metabolite abundance patterns.

**Figure 4:**
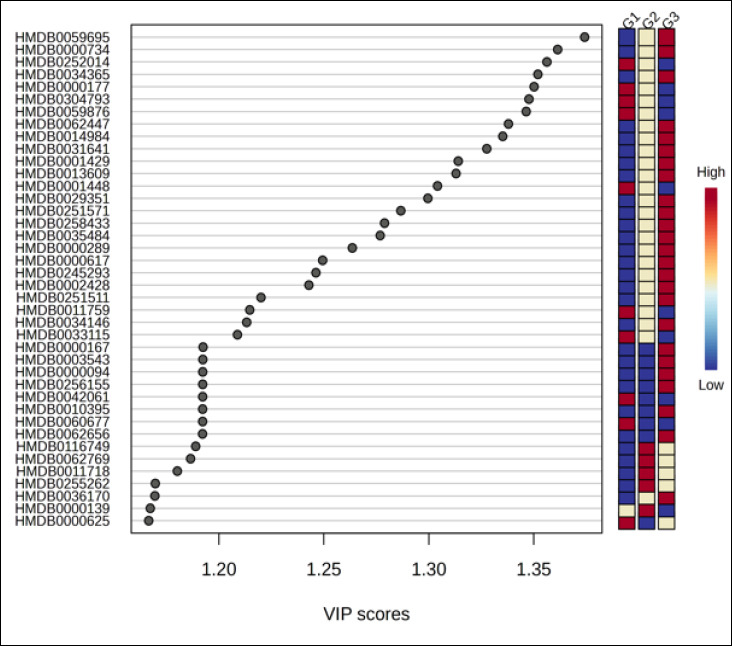
Partial least squares–discriminant analysis variable importance in projection score plot of differential metabolites in Alope village chickens across age groups.

Univariate screening supported these findings. The ANOVA bubble plot (Figure 3A) demonstrated numerous features with strong evidence of group differences, indicated by higher −log_10_p values and larger effect magnitudes across the m/z–retention-time space. Similarly, hierarchical clustering analysis (Figure 3B) clearly separated samples by age and revealed coherent metabolite modules that increased monotonically from G1 to G3 or declined in the opposite direction, with G2 exhibiting an intermediate, transitional profile. The VIP score plot (Figure 4) ranked the metabolites contributing most strongly to class separation (VIP ≈ 1.2–1.37), with accompanying heat strips showing consistent age-related abundance gradients. Collectively, these analyses demonstrate robust, system-level remodeling of the plasma metabolome with age in Alope chickens and define a focused subset of metabolites for downstream pathway analysis and validation.

### Pathway-level metabolite enrichment

Pathway enrichment analysis was performed in an exploratory manner to contextualize age-associated metabolite differences at the pathway-level. Twelve KEGG pathways contained at least one matched metabolite; however, none reached statistical significance after false discovery rate correction (all FDR ≥ 0.35). Accordingly, pathway-level findings are reported descriptively as associative patterns rather than causal inferences. The glycine, serine, and threonine metabolism pathway exhibited the highest topology impact (0.331; hits = 2: HMDB0000167, HMDB0000139) and moderate signal intensity on the bubble plot (−log_10_p ≈ 1–1.2). Glycerolipid metabolism (impact = 0.093; HMDB0000139) and the citrate cycle (impact = 0.090; HMDB0000094) also showed non-trivial impacts based on single metabolite hits. The pentose phosphate and glyoxylate/dicarboxylate pathways each included two hits (FDR = 0.347) but exhibited low impact scores (≤ 0.042 and 0.010, respectively). Additional single-hit associations were observed for histidine, purine, glycerophospholipid, β-alanine, and branched-chain amino acid biosynthesis pathways (FDR = 0.62–1.00). Overall, these results suggest that age-associated metabolic variation is primarily linked to amino acid metabolism, with secondary contributions from lipid and central energy-related processes.

### Biomarker candidate identification

Supervised multivariate modeling identified seven putative biomarker candidates with high discriminatory contribution (VIP ≈ 1.17–1.35) and strong single-analyte classification performance (AUC ≥ 0.985). These candidates were derived from internal model optimization and should therefore be considered hypothesis-generating rather than definitive diagnostic markers. Three metabolites characterized the youngest age group (G1): HMDB0000625, HMDB0001448, and HMDB0000177. HMDB0000139 peaked at mid-growth (G2), whereas HMDB0000094, HMDB0000167, and HMDB0000289 were enriched in the oldest group (G3), collectively reflecting age-related shifts in amino acid, purine, and carbohydrate–lipid metabolism. Group-wise boxplots demonstrated minimal overlap among age categories, supporting monotonic age-associated abundance trends. Despite strong internal discrimination, external validation using independent and preferably longitudinal cohorts is required to confirm robustness and minimize overfitting. From a practical standpoint, several of these metabolites are chemically stable and analytically accessible, supporting their potential use in future targeted assays and positioning them as discovery-level leads for age-informed metabolic indicators in village chicken production systems.

## DISCUSSION

### Age-associated metabolic reorganization

**Figure 5:**
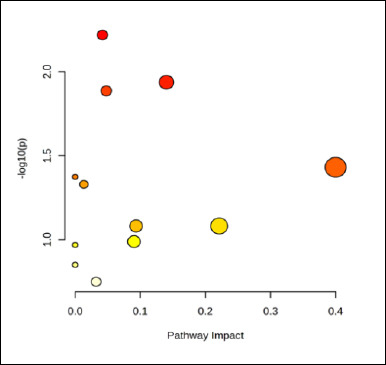
Bubble plot of metabolite-associated pathways in Alope village chickens across age groups.

Age emerged as the dominant axis structuring metabolism in Alope chickens. Across the 8–16-week growth window, concordant changes in blood biochemistry and plasma metabolomics indicate coordinated metabolic reorganization with growth rather than isolated shifts detected by either approach alone. The progressive increases in glucose, total protein, urea, and triglycerides observed in Figure 1 are consistent with a developmental transition from early lean tissue accretion toward greater emphasis on lipid handling and a more stable energy balance at later stages, with no evidence of a metabolic plateau within the studied age range [17, 18]. Multivariate projections clearly separated age groups in the PCA score plot and identified the metabolites driving this separation in the PLS-DA biplot (Figure 2), while univariate screening and hierarchical clustering revealed coordinated metabolite modules that increased or declined with age (Figure 3) [19,20]. Pathway-level trends supported these findings, with contributions from glycine–serine–threonine metabolism, glycerolipid and glycerophospholipid metabolism, purine metabolism, the citrate cycle, and nodes linked to the pentose phosphate pathway (Figure 5, Table 1) [21]. In the avian context, enrichment labels are best interpreted as topological descriptors of shared metabolic nodes rather than literal pathway activation, consistent with the dicarboxylate–tricarboxylic acid cycle. intersections suggested here [22].

**Table 1:** Exploratory pathway enrichment analysis of plasma metabolites across age groups in Alope village chickens.

Pathway	Total	Hits	FDR	Impact	Metabolite(s)
The pentose phosphate pathway	23	2	0.34681	0.04185	HMDB0000625, HMDB0000139
Glyoxylate and dicarboxylate metabolism	32	2	0.34681	0.00972	HMDB0000139, HMDB0000094
Glycine, serine, and threonine metabolism	34	2	0.34681	0.33125	HMDB0000167, HMDB0000139
Sulfur metabolism	7	1	0.62488	0.00278	HMDB0001448
Biosynthesis of valine, leucine, and isoleucine	8	1	0.62488	0.00000	HMDB0000167
Purine metabolism	67	2	0.62488	0.01326	HMDB0000289, HMDB0001448
Glycerolipid metabolism	16	1	0.82987	0.09346	HMDB0000139
Histidine metabolism	16	1	0.82987	0.22131	HMDB0000177
Citrate cycle (TCA cycle)	20	1	0.86123	0.09038	HMDB0000094
β-Alanine metabolism	21	1	0.86123	0.00000	HMDB0000177
Alanine, aspartate, and glutamate metabolism	28	1	1.00000	0.00000	HMDB0000177
Glycerophospholipid metabolism	36	1	1.00000	0.03208	HMDB0000177

Pathway enrichment results are presented as exploratory associations. Total indicates the total number of metabolites mapped to each pathway, Hits represent the number of detected metabolites mapped to the pathway, FDR denotes false discovery rate–adjusted p value, Impact reflects pathway topology impact based on network position, HMDB = Human Metabolome Database, TCA = Tricarboxylic acid cycle.

Pathway enrichment results are presented as exploratory associations. Total indicates the total number of metabolites mapped to each pathway, Hits represent the number of detected metabolites mapped to the pathway, FDR denotes false discovery rate–adjusted p value, Impact reflects pathway topology impact based on network position, HMDB = Human Metabolome Database, TCA = Tricarboxylic acid cycle.

### Functional interpretation of biomarker candidates

**Figure 6:**
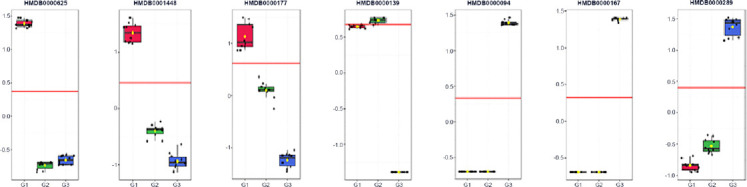
Receiver operating characteristic boxplot of metabolite biomarker candidates in Alope village chickens across age groups.

Integration of biochemical and metabolomic layers enabled translation of these findings into stage-relevant nutritional implications. The seven putative biomarker candidates identified (Figure 6, Table 2) mapped consistently to three functional axes, namely one-carbon/redox economy, membrane-lipid turnover, and nucleotide/energy metabolism, reflecting the biochemical trajectories observed across ages. In the youngest age group, HMDB0000625 (pentose phosphate pathway), HMDB0001448 (sulfur and purine metabolism), and HMDB0000177 (histidine, β-alanine, and glycerophospholipid pathways) collectively indicate elevated requirements for NADPH generation, antioxidant and methyl-group capacity, histidine–carnosine buffering, and active membrane remodeling. From a nutritional standpoint, this stage would benefit from higher digestible protein density with balanced essential amino acids, adequate methionine and cysteine supply, sufficient glucogenic substrates to support one-carbon flux, and controlled lipid provision favoring membrane biogenesis over storage [23].

**Table 2:** Plasma metabolite biomarker candidates discriminating age groups in Alope village chickens.

HMDB ID	Direction	VIP	Pathway	AUC
HMDB0000625	G1	1.1666	The pentose phosphate pathway	1.000
HMDB0000139	G2	1.1673	Pentose phosphate pathway, Glyoxylate and dicarboxylate metabolism, Glycine, serine, and threonine metabolism, Glycerolipid metabolism	0.985
HMDB0000094	G3	1.1923	Glyoxylate and dicarboxylate metabolism	1.000
HMDB0000167	G3	1.1925	Glycine, serine, and threonine metabolism, Biosynthesis of valine, leucine, and isoleucine	1.000
HMDB0001448	G1	1.3042	Sulfur metabolism, Purine metabolism	1.000
HMDB0000289	G3	1.2636	Purine metabolism	1.000
HMDB0000177	G1	1.3502	Histidine metabolism, β-Alanine metabolism, Glycerophospholipid metabolism	1.000

Direction indicates the age group (G1, G2, or G3) in which the metabolite showed the highest relative abundance, VIP denotes variable importance in projection score derived from partial least squares–discriminant analysis, Pathway refers to the primary metabolic pathways associated with each metabolite, AUC represents the area under the receiver operating characteristic curve indicating classification performance, HMDB = Human Metabolome Database.

Direction indicates the age group (G1, G2, or G3) in which the metabolite showed the highest relative abundance, VIP denotes variable importance in projection score derived from partial least squares–discriminant analysis, Pathway refers to the primary metabolic pathways associated with each metabolite, AUC represents the area under the receiver operating characteristic curve indicating classification performance, HMDB = Human Metabolome Database.

At the intermediate growth stage, HMDB0000139 showed peak abundance and bridged pentose phosphate, glycine–serine–threonine, glyoxylate–dicarboxylate, and glycerolipid metabolism, suggesting a transitional redistribution of carbon toward membrane assembly and one-carbon trafficking. At this stage, diets should maintain a balanced energy-to-protein ratio, ensure adequate glycine and serine availability, and provide choline and essential fatty acids to support phospholipid turnover without promoting excessive neutral-lipid deposition [4]. In the oldest chickens, enrichment of HMDB0000094 (glyoxylate–dicarboxylate metabolism), HMDB0000167 (glycine–serine–threonine metabolism with links to branched-chain amino acids), and HMDB0000289 (purine metabolism) reflects increased mitochondrial flux and anaplerosis, reorganization of amino acid utilization as growth shifts toward maintenance, and heightened nucleotide turnover approaching physiological maturity. Nutritional strategies at this stage should emphasize slightly higher energy density with careful control of fat quality, continued amino acid balance with attention to threonine and branched-chain profiles, and micronutrients supporting redox balance and nucleotide economy, with field validation recommended prior to routine application [24].

### Study limitations and future perspectives

Several limitations should be considered when interpreting these findings. First, the study focused on a single village chicken variety with a modest sample size (n = 10 per age group), limiting statistical power and generalizability for omics-based inference [25]. Second, the cross-sectional design captured population-level age differences rather than individual developmental trajectories, and the restricted age window of 8–16 weeks precludes inference beyond this period [26]. Third, the metabolomics platform provided partial chemical coverage biased toward derivatizable compounds, and pathway enrichment signals did not withstand conservative multiple-testing correction, rendering pathway associations exploratory rather than confirmatory [27]. Fourth, UA, the principal nitrogenous waste product in birds, was not measured, and endocrine indicators of growth and metabolism, including insulin, thyroid hormones, and corticosterone, were not assessed, limiting resolution of nitrogen handling and hormonal regulation [28]. Finally, environmental and management variability typical of smallholder systems was not explicitly modeled and may have influenced metabolic profiles [29].

### Implications for village chicken production systems

Taken together, Figures 1–6 and Tables 1–2 delineate a coherent trajectory of metabolic maturation in Alope chickens, in which early growth is characterized by elevated one-carbon/redox and membrane synthesis demands, mid-growth reflects transitional substrate redistribution, and later growth emphasizes mitochondrial energy handling and nucleotide turnover. Although these integrated biochemical and metabolomic patterns provide a robust descriptive framework, their application to precision feeding or routine physiological monitoring should be regarded as provisional, pending validation in larger, longitudinal cohorts under controlled dietary and environmental conditions. Notably, this study establishes age-stratified biochemical reference values for an indigenous village chicken genotype, integrates field-relevant clinical chemistry with untargeted plasma metabolomics, and introduces physiological age as a metabolomics-informed construct rather than relying solely on chronological age. By linking metabolic maturation to nutrition-sensitive pathways, this work contributes to sustainable poultry production and One Health–aligned food systems in low- and middle-income settings.

## CONCLUSION

This study demonstrates that age is the primary determinant of metabolic organization in Alope chickens during the 8–16-week growth period. Progressive and significant increases in glucose, total protein, urea, and triglycerides (*p* < 0.05) indicate a coordinated physiological transition from early lean tissue accretion toward enhanced lipid handling and more stable energy balance. Concordant plasma metabolomic profiling revealed clear age-dependent clustering, coherent metabolite modules, and pathway-level shifts primarily involving amino acid metabolism, lipid turnover, purine metabolism, and central energy pathways. Seven putative age-discriminant metabolites were identified, showing strong internal discriminatory performance and reflecting coordinated changes in one-carbon/redox balance, membrane remodeling, and nucleotide and energy metabolism.

The integration of blood biochemistry with plasma metabolomics provides a systems-level framework for defining physiological age in Alope chickens beyond chronological measures alone. The identified biochemical trends and metabolite candidates support age-informed nutritional strategies, including higher digestible protein density and redox support during early growth, balanced energy–protein supply with phospholipid support during mid-growth, and controlled energy density with optimized fat quality and amino acid balance at later stages. These insights have direct relevance for improving feeding efficiency, physiological monitoring, and sustainability in village and smallholder chicken production systems.

Key strengths include the integration of field-relevant clinical biochemistry with untargeted plasma metabolomics, the establishment of age-stratified biochemical reference patterns for an indigenous village chicken genotype, and the identification of functional biomarker axes linked to nutrition-sensitive metabolic processes rather than production traits alone. The consistent agreement between univariate, multivariate, and pathway-level analyses reinforces the biological coherence of the findings.

Several limitations should be acknowledged. The study was conducted on a single village chicken variety with a modest sample size, limiting generalizability. The cross-sectional design captured population-level age differences rather than individual developmental trajectories, and the metabolomics platform provided partial chemical coverage. In addition, pathway enrichment results were exploratory, UA and endocrine regulators were not measured, and environmental variability typical of smallholder systems was not explicitly modeled.

Future research should validate these findings in larger and independent cohorts, preferably using longitudinal designs that track individual metabolic trajectories. Targeted quantification of candidate metabolites, integration with hormonal and genetic data, and evaluation under controlled dietary and environmental interventions will be essential to confirm robustness and translational value. Expansion of this framework to other village chicken populations may further enhance its applicability.

In conclusion, this study provides an integrated biochemical and metabolomic framework for understanding age-related metabolic maturation in Alope chickens (*G. g. domesticus*). By linking physiological age to nutrition-sensitive metabolic pathways, the findings advance precision nutrition concepts into low-input and smallholder poultry systems and contribute to sustainable production strategies aligned with One Health principles.

## DATA AVAILABILITY

The raw GC–MS data and the processed metabolomics datasets generated during the current study are not publicly deposited but are available upon reasonable request from the corresponding author. Access to the data is subject to institutional policies and ethical considerations and will be provided for academic and non-commercial research purposes to support transparency and reproducibility.

## AUTHORS’ CONTRIBUTIONS

AM, AMD, MRB, MFAKW, AN, HH, and SS: Conceived, designed, and coordinated the study. AM, MRB, MFAKW, APB, AN, and FAP: Contributed and were the principal investigators. AM, MRB, MFAKW, APB, AN, FAP, and WP: Supervised field sampling, data collection, laboratory work, and data entry. AM, AMD, MRB, and AN: Statistical analysis and interpretation and drafted the manuscript. All authors have read and approved the final version of the manuscript.

## COMPETING INTERESTS

The authors declare that they have no competing interests.

## PUBLISHER’S NOTE

Veterinary World remains neutral with regard to jurisdictional claims in the published institutional affiliations.
